# Nitrogen Balance and Protein Requirements for Critically Ill Older Patients

**DOI:** 10.3390/nu8040226

**Published:** 2016-04-18

**Authors:** Roland N. Dickerson

**Affiliations:** Department of Clinical Pharmacy, University of Tennessee College of Pharmacy, 881 Madison Avenue, Suite 345, Memphis, TN 38163, USA; rdickerson@uthsc.edu; Tel.: +1-901-448-6420

**Keywords:** nitrogen balance, critical illness, requirements, trauma, protein, aging, elderly, catabolism, obesity

## Abstract

Critically ill older patients with sarcopenia experience greater morbidity and mortality than younger patients. It is anticipated that unabated protein catabolism would be detrimental for the critically ill older patient. Healthy older subjects experience a diminished response to protein supplementation when compared to their younger counterparts, but this anabolic resistance can be overcome by increasing protein intake. Preliminary evidence suggests that older patients may respond differently to protein intake than younger patients during critical illness as well. If sufficient protein intake is given, older patients can achieve a similar nitrogen accretion response as younger patients even during critical illness. However, there is concern among some clinicians that increasing protein intake in older patients during critical illness may lead to azotemia due to decreased renal functional reserve which may augment the propensity towards worsened renal function and worsened clinical outcomes. Current evidence regarding protein requirements, nitrogen balance, ureagenesis, and clinical outcomes during nutritional therapy for critically ill older patients is reviewed.

## 1. Introduction

Critical illness is associated with hypermetabolism and marked protein catabolism [1,2]. When excessive protein catabolism is left unabated, patients can experience decreased immunity, increased infections, and worsened survival [3,4,5,6]. Critically ill, older surgical or trauma patients with sarcopenia experience greater mortality, more post-operative complications, decreased ventilator-free days and decreased intensive care unit (ICU)-free days [7,8]. It is well established that older patients have less muscle mass and more fat mass than their younger counterparts of similar body weight [9]. Muscle erosion typically begins after 55 years of age and it has been estimated that the cumulative decline in muscle mass reaches approximately 30% to 50% by 80 years of age when compared to those 20 years old [10]. Given the pre-existing depletion of muscle mass, it is anticipated that unabated protein catabolism would be detrimental for the critically ill older patient. Therefore, it is important to appropriately identify who may be at risk for poorer clinical outcomes and who may benefit from an aggressive nutritional strategy.

## 2. Nutritional Assessment of Older Patients

Conventional methods for nutrition assessment are problematic and frequently fail in identifying the “at-risk” critically ill older patient. Although a low body mass index (BMI) has been associated with higher mortality rates in older patients [11], the relative risk of death from being overweight declines with age [12,13]. Disability-free years and highest life expectancy is greatest among healthy older subjects with a BMI of 25 to 30 kg/m^2^ [14]. The lowest risk of mortality for hospitalized older patients in one study occurred at a BMI of about 30 kg/m^2^ [15]. A major difficulty encountered in clinical practice is the inability to accurately identify the presence of sarcopenia. Sarcopenia has been objectively defined as an appendicular skeletal muscle mass/height^2^ less than two standard deviations below the mean for young, healthy reference standards [16]. However, computed tomography scans at the third lumbar region for estimation of whole body muscle and adipose tissue mass distribution [7] are not readily available to most clinicians. Anthropometric measurements are generally unreliable in the intensive care unit, even with efforts to reduce operator measurement error. The unreliability of anthropometric measurements is further compounded with aging as older patients experience a decreased ability to excrete a water load and prolonged overexpansion of extracellular water following resuscitation or sepsis [17,18]. Additionally, inflammation and critical illness render serum proteins including serum prealbumin concentration unreliable [19].

The European Working Group on Sarcopenia in Older People recommend assessing for the presence of decreased muscle mass and low muscle function (strength and performance) [20]. However, due the difficulty in evaluating these indicators in the ICU environment, most clinicians empirically assess the patient based on physical exam combined with family, social, and medical history. A heightened suspicion for sarcopenia is assumed based on irregularities in the physical exam and patient histories. A presumed assumption of sarcopenia is accepted if the patient has a history of signs and symptoms of frailty since most frail patients have sarcopenia [20]. Frailty has been associated with increased incidence of falls, worsening of mobility or activities of daily living disability, hospitalization, and death. Frailty may be defined as having three of more of the following criteria: unintentional weight loss of 10 pounds or more in the past year, self-reported exhaustion, weakness, slow walking speed, or low physical activity [21]. Intermediate frailty status, as indicated by the presence of one or two of these criteria, indicated intermediate risk for these detrimental outcomes as well as increased risk for becoming frail over the next 3 to 4 years [21]. The importance of frailty upon determination of a nutritional plan is paramount as frailty exacerbates age-related changes in protein metabolism by inducing an increase in muscle protein catabolism and a decrease in muscle mass [22].

## 3. Determination of Protein Requirements in Clinical Practice

Existing guidelines for current dietary protein intake recommendations for recommended dietary reference intake (DRI) or recommended dietary allowance (RDA) for adults is based on nitrogen balance studies [10]. The concept of nitrogen balance is that the difference between nitrogen intake and loss reflects gain or loss of total body protein. If more nitrogen (protein) is given to the patient than lost, the patient is considered to be anabolic or “in positive nitrogen balance”. If more nitrogen is lost than given, the patient is considered to be catabolic or “in negative nitrogen balance”. A nitrogen balance within −4 or −5 g/day to +4 or +5 g/day is usually considered “nitrogen equilibrium”. However, it is important to note that nitrogen balance reflects only the net result of nitrogen exchange. It does not give insight into the dynamics of protein synthesis or catabolism or subtle changes in protein redistribution (e.g., shifts between muscle, splanchnic tissue, and other organ systems).

From the practical standpoint, determination of nitrogen balance has its limitations. A nitrogen balance study requires accurate determination of protein intake and a precise accounting of all sources of nitrogen excretion. The most popular method for estimating nitrogen balance used in clinical practice assumes that total nitrogen loss is equal to urinary urea nitrogen excretion and an additional constant loss of 4 g/day [23]. The constant factor of 4 g/day makes the assumption that 2 g of nitrogen loss is derived from non-urinary urea nitrogen loss since most hospitals can only measure urea nitrogen and not total urinary nitrogen. The other 2 g of loss out of the 4 g is from integumentary, gastrointestinal and insensible losses. However, these assumptions underestimate non-urea urinary nitrogen (e.g., ammonia, creatinine, uric acid, amino acids) for catabolic critically ill patients [24], gastrointestinal losses for those with diarrhea [25], and integumentary losses in patients with thermal injuries [26]. Finally, it is unlikely that a steady-state nitrogen balance determination can be achieved for most critically ill patients due to interruptions in enteral nutrient delivery and day-to-day fluctuations in the patients’ clinical status. Taken together, these limitations may ultimately result in an underestimation of nitrogen losses and protein requirements.

The rationale for use of nitrogen balance as a marker for adequate protein intake may be questioned due to the lack of large, randomized prospective trials examining nitrogen balance-guided protein intake upon clinical outcomes for critically ill patients. However, limited evidence from observational studies suggest increasing protein intake above normal maintenance requirements with improvement in nitrogen balance may be of benefit for critically ill patients. One prospective, observational cohort study in 113 critically ill surgical and medical intensive care unit patients suggested a higher mean protein intake of 1.5 g/kg/day *vs.* 1.1 g/kg/day or 0.8 g/kg/day led to a significantly improved mean nitrogen balance of −2.6 g/day *vs.* −4.6 g/day *vs.* −6.6 g/day as well as a trending improvement in intensive care unit mortality (16% *vs.* 24% and 27%, respectively) [27]. In a prospective randomized design, 50 critically ill patients with acute kidney injury who required continuous renal replacement therapy received either 1.5, 2, or 2.5 g/kg/day of protein with a caloric intake designed to match measured or predicted energy expenditure. The investigators found that nitrogen balance was improved by increasing protein intake and for every 1 g/day increase in nitrogen balance, the probability of survival increased (odds ratio of 1.21, *p* = 0.03) [28]. Further research regarding the role of nitrogen balance relative to clinical outcomes in acutely ill, hospitalized patients is clearly needed.

## 4. Protein Requirements for Older Adults

### 4.1. Requirements of Healthy Older Subjects

Although the estimated mean protein requirement of healthy individuals is defined as 0.8 g/kg/day by the Food and Nutrition Board of the U.S. National Research Council, it has been suggested that elderly subjects may require more protein than younger individuals [10,16,29,30]. It is apparent that healthy elderly subjects have a diminished ability to mount a similar anabolic response to protein supplementation compared to younger patients [10,16,31,32]. Despite a higher splanchnic extraction of orally administered amino acids in older subjects, increased muscle protein synthesis can be still be elicited by increasing amino acid or protein intake [33]. The etiology for this diminished anabolic response is not entirely clear and likely multifactorial [31,33,34,35]. Current data indicates that the “anabolic resistance” associated with aging can be overcome if sufficient protein intake, particularly if supplemental leucine [36,37] or beta hydroxy-methylbutyrate [38] is given to stimulate muscle protein synthesis [16,31,33,34,39,40]. It has been suggested that a safe protein intake for healthy elderly adults would be 1 to 1.25 g/kg/day of high quality protein [29]. However, some experts argue that there is insufficient evidence to establish a firm recommendation regarding protein requirements for older healthy subjects [10].

### 4.2. Hypocaloric, High Protein Nutrition Therapy for Critically Ill Older Patients with Obesity

Unfortunately, there are no randomized controlled trials and a minimal number of observational studies examining the protein needs of critically ill older patients despite the need to ascertain if “the anabolic resistance” of aging can be overcome with higher protein intake under these catabolic conditions [41,42,43]. Recent guidelines, based on expert opinion with relatively few supporting observational studies, recommend the use of hypocaloric, high protein nutrition therapy for the acutely ill, hospitalized patient with obesity [44,45]. The intent of this mode of nutritional therapy is to achieve an anabolic effect while potentially prevent complications of overfeeding (e.g., hypercapnia, hyperglycemia, fatty infiltration of the liver) in a high risk population whereby energy needs are difficult to assess and who may already have insulin resistance, glucose intolerance, non-alcoholic fatty liver disease and hypoventilation syndrome [44]. A higher protein intake is suggested with hypocaloric feeding in an effort to achieve the same anabolic effect upon nitrogen balance as a higher calorie, lower protein regimen [46,47]. Critically ill patients with obesity who receive a hypocaloric nutrition regimen may require protein intakes of 2 to 2.5 g/kg ideal body weight/day and non-ICU patients may require 1.8 to 1.9 g/kg ideal body weight/day to approach nitrogen equilibrium [48]. However, it should be noted that this type of nutrition therapy also results in decreased net protein utilization and a modest increase in ureagenesis [42,46,47].

The first observational study examining the use of hypocaloric, high protein parenteral nutrition therapy in hospitalized older patients with obesity retrospectively evaluated 18 patients <60 years of age and 12 patients 60 years of age or older [43]. Patients received parenteral nutrition for an average of 13 days. Both groups received 18 kcal/kg actual weight/day. Protein intakes were similar between age groups at ~1.8 ± 0.4 g/kg ideal body weight/day and ~1.9 g/kg ideal body weight/day, respectively. Nitrogen balance tended to be greater for the younger group: 3.4 ± 3.9 g/day *vs.* 0.2 ± 5.0 g/day (*p* = 0.06). Only one patient from the younger group experienced a negative nitrogen balance compared to 5 patients in the older group (*p* = 0.025). Unfortunately, serum urea nitrogen concentrations and clinical outcomes were not examined. The authors concluded that the older obese patients may continue to undergo protein catabolism with hypocaloric, high-protein nutrition therapy and that this form of nutritional support should be used with caution in older patients.

**Figure 1:**
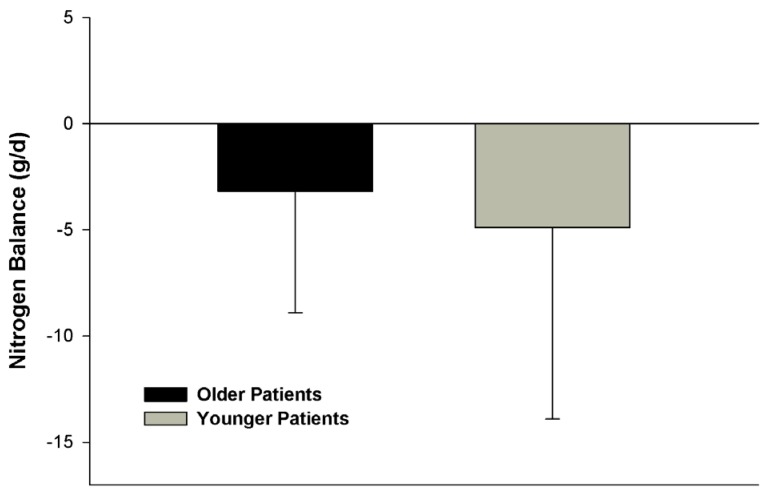
Nitrogen balance determination during hypocaloric high protein nutrition therapy between older and younger critically ill patients with traumatic injuries and obesity. No significant difference in nitrogen balance was noted between groups (*p* = 0.363). Reprinted with permission from Dickerson, R.N.; Medling, T.L.; Smith, A.C.; Maish, G.O., 3rd,; Croce, M.A.; Minard, G.; Brown, R.O. Hypocaloric, high-protein nutrition therapy in older *vs.* younger critically ill patients with obesity. *JPEN J. Parenter. Enter. Nutr.* **2013**, *37*, 342–351. A.S.P.E.N. does not endorse the use of this material in any form other than its entirety.

We presumed the trending difference in nitrogen balance between age groups in the above study [43] may have been attributed to potential anabolic resistance associated with aging. Conversely, we hypothesized that an insufficient amount of protein, particularly in reference to caloric intake, was given to overcome this resistance in that study. To test this hypothesis, we retrospectively compared the metabolic response to hypocaloric high protein nutrition therapy in 33 older (60 years of age and older) and 41 younger (18 to 59 years of age) with obesity and traumatic injuries [42]. Only patients who received at least 2 g/kg ideal body weight/day of protein intake and a nitrogen balance determination at goal protein intake were enrolled for study. When given isonitrogenous regimens (2.3 ± 0.3 g/kg ideal body weight/day *vs.* 2.3 ± 0.2 g/kg ideal body weight/day, respectively) and at a greater protein intake than the Liu study [43], no significant difference in nitrogen balance was noted between age groups (Figure 1). Although it was anticipated that the majority of patients in our study would be in negative nitrogen balance due to critical illness and timing of the nitrogen balance determination post-traumatic injury, about half of the patients from each group achieved a positive nitrogen balance or nitrogen equilibrium. These data suggested that the severity of the net protein catabolism could be at least partially ameliorated by aggressive nutrition therapy [1].

Clinical outcomes, including survival, duration of ICU stay, duration of mechanical ventilation, duration of hospital stay, or incidence of infectious complications, were not different between age groups. However, the sample size of this study was likely insufficient to ascertain a difference in clinical outcomes. It was concluded that older patients had an equivalent nitrogen balance response to younger patients when given adequate protein intake and refutes previously published data that suggested older patients with obesity cannot overcome anabolic resistance associated with aging during hypocaloric, high protein nutrition therapy [43].

### 4.3. Comparative Nitrogen Accretion Response to Protein Intake in Older vs. Younger Non-Obese Patients with Severe Traumatic Injuries

Since marked protein catabolism occurs following traumatic injury, achievement of nitrogen equilibrium or positive nitrogen balance was only possible in only about half of the non-obese patients despite receiving an aggressive protein intake of 2 to 2.5 g/kg/day [1]. Because it was unclear as to what extent “anabolic resistance” associated with aging played a role in determination of protein requirements during critical illness, we retrospectively examined nitrogen balance determinations in 54 older (≥60 years of age) and 195 younger (18 to 59 years of age) non-obese patients with traumatic injuries [41]. Nitrogen balance determinations were conducted, as part of the patient’s routine metabolic care, 5 to 14 days post-admission to the trauma intensive care unit during the “flow phase” of injury.

**Figure 2:**
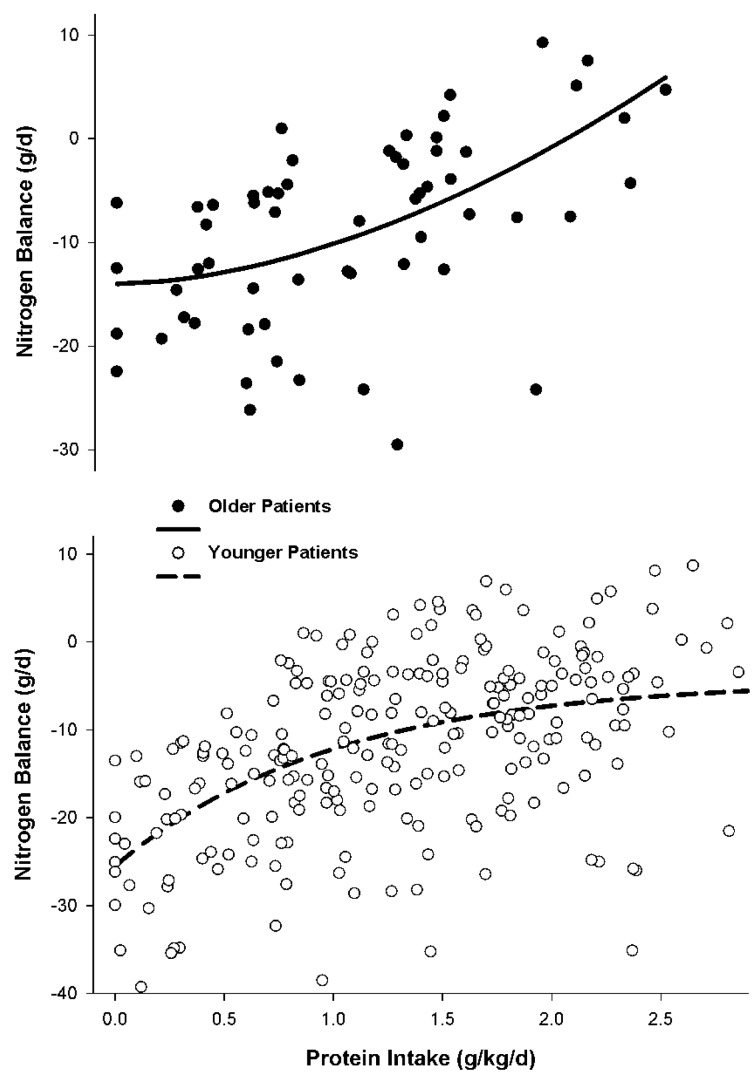
Nonlinear relationship between protein intake on nitrogen accretion for older and younger patients. Nonlinear correlative relationships were significant (*p* < 0.001) between nitrogen balance and protein intake were r = 0.51 and r = 0.50 for the older and younger patients, respectively. Reprinted with permission from Dickerson, R.N.; Maish, G.O., 3rd; Croce, M.A.; Minard, G.; Brown, R.O. Influence of aging on nitrogen accretion during critical illness. *JPEN J. Parenter. Enter. Nutr.* **2015**, *39*, 282–290. A.S.P.E.N. does not endorse the use of this material in any form other than its entirety.

The data indicated considerable variability in nitrogen balance response to varying protein intakes among both older and younger patient groups. It was evident that a blunted improvement in nitrogen accretion in response to lower protein intakes occurred in older patients when compared to younger patients during critical illness (Figure 2). Older patients exhibited a concave relationship between protein intake and nitrogen balance response. Minimal change in nitrogen balance occurred at protein intakes less than 1.5 g/kg/day for older patients. However, when protein intakes of 1.5 to 2.5 g/kg/day were given, nitrogen accretion significantly improved (Figure 2). Conversely, in younger patients, a convex relationship between protein intake and nitrogen balance was demonstrated. Progressive improvement in nitrogen accretion occurred as protein intake increased until a protein intake of ~1.7 g/kg/day to 2.2 g/kg/day was achieved whereby higher doses resulted in only minor incremental improvements in nitrogen balance (Figure 2).

Close inspection of Figure 2 also depicts a potential difference in the severity of catabolism at baseline (protein intake of 0 g/kg/day) based on nitrogen balance between the age groups. Older patients experienced about half the extent of hypercatabolism as younger patients at a nitrogen balance of −13 g/day as opposed to about −25 g/day for the younger patients. Previous data indicated that increases in total body weight appear inversely associated with worsening nitrogen balance in trauma patients [1]. Median body weights between older and younger patients groups were the same (80 kg) in our evaluation of nitrogen accretion response in older *versus* younger patients. However, since the younger patients would be expected to have more muscle mass it is possible that the younger patients may exhibit greater urinary nitrogen losses than older patients due to greater availability of protein substrate. Conventional assessment markers for assessing for differences in the severity of their illness between the age groups, including body temperature, white blood cell count, ventilator dependency, presence of sepsis, ICU length of stay, hospital length of stay, and serum glucose concentration, were not predictive for these differences in baseline nitrogen catabolism [41]. Median injury severity score was lower in the older patients compared to younger patients (26 *vs.* 30) and a greater amount of younger patients had traumatic brain injury than older patients (37% *vs.* 20% of patients). However, it was unlikely that these variables attributed to the observed differences in baseline nitrogen catabolism as it has been previously established that injury severity score and the presence of traumatic brain injury are unreliable predictors of extent of protein catabolism and urinary nitrogen excretion [1,49]. Etiologies for this difference in the baseline level of protein catabolism following traumatic injury between age groups requires further study.

These data suggest older and younger critically ill patients with traumatic injuries respond differently to varying increases in protein intake. It is also clear that there is considerable variability in nitrogen balance response to incremental increases in protein intake among both older and younger patients (Figure 2). Randomized controlled trials to investigate the best way to guide individualization of protein intake and subsequent assessment of adequacy of the prescribed intake is warranted. Until these studies are available, one empiric method to individualization is to use serial nitrogen balance measurements and serum urea nitrogen concentrations to guide protein intake. Our approach for our critically ill patients with traumatic injuries is to adjust protein intake in an effort to achieve near nitrogen equilibrium (e.g., nitrogen balance of −4 g/day to +4 g/day) if possible. We employ a ceiling protein dose of about 2.5 to 3 g/kg/day for our highly catabolic trauma patient population [1,41]. Protein dosage may be guided by lack of improvement in nitrogen balance and/or the presence excessive ureagenesis [1]. A substantial increase in serum urea nitrogen concentration in the absence of renal impairment or dehydration suggests augmented ureagenesis also implying futility in the current protein dosage [1,41].

## 5. Impact of Protein Intake upon Renal Function in Older Patient

### 5.1. Glomerular Filtration Rate, Creatinine Clearance and Renal Functional Reserve

One of the common concerns expressed by clinical practitioners about providing higher protein intakes to older critically ill adults is their increased risk for decreased kidney function and reduced glomerular filtration rate [9]. Aging is associated with a loss of renal mass [50] and longitudinal studies suggest aging is associated with a decline in glomerular filtration rate [51]. Mean glomerular filtration rate (measured by inulin clearance) declines from about 120 mL/min/1.73 m^2^ at 40 years of age to 90 mL/min/1.73 m^2^ by 60 years of life [52]. When protein intake is restricted or reduced in normal subjects, urinary urea excretion and creatinine clearance is reduced [53]. As protein intake increases, urinary urea nitrogen excretion and creatinine clearance increases, but ultimately a plateau in creatinine clearance is anticipated [53]. These changes are not due to the creatinine content of the ingested protein as only about 10% of daily urinary creatinine excretion is from exogenous origin for non-vegetarians and is not a consideration for patients receiving commercial enteral formulations or parenteral nutrition [54]. As a result, it has been argued that the age-related fall in glomerular filtration rate, in the absence of diseases known to cause renal dysfunction such as diabetes mellitus and hypertension, is due to a decreased dietary intake of protein [53]. Conversely, it also has been argued that higher protein intakes may predispose the patient with chronic kidney disease to progression of their kidney disease and earlier onset of hemodialysis; however, data to support this hypothesis is conflicting [55,56].

Part of the difficulty in assessing renal function in older adults is that the primary source of urinary creatinine excretion is muscle mass which is decreased in older adults. Thus a normal serum creatinine concentration in an older patient does not accurately reflect glomerular filtration rate and also explains why conventional methods to calculate predicted creatinine clearance incorporates both age and sex into the equations [57]. Others have argued that estimation of creatinine clearance by the Cockcroft-Gault equations may actually underestimate glomerular filtration rate and the clearance of drugs that are excreted by the kidney in older healthy subjects [58]. Other markers of renal function such as cystatin C which may be more accurate than creatinine in the elderly [59], but are not routinely available in many hospital laboratories.

The concerning hypothesis for reluctance towards providing an increased protein intake is that the renal vasodilatory response to an amino acid load, also referred to as renal functional reserve, may be compromised in the elderly. The net response, given a blunted or absent renal functional reserve and increased protein intake, would be a marked increase in serum urea nitrogen concentration, increased prevalence of azotemia, and possibly uremia. Fliser and colleagues compared markers of renal functional reserve before and after an intravenous amino acid infusion (~0.7 g/kg over 8 h) in younger (mean of 26 years of age) and older (mean age of 70 years) healthy subjects [52]. Although baseline glomerular filtration rate and effective renal plasma flow were slightly lower in the older patients, inulin clearance, reflective of glomerular filtration rate, increased by 16% and 17% after the amino acid infusion for the younger and older subjects, respectively. Their work indicated that renal functional reserve is well up preserved at least until 80 years of life [52].

The decrease in glomerular filtration rate that occurs with aging is generally much less than necessary to elicit symptoms of renal failure [9]. This observation is further supported by recent data from the Cardiovascular Health Study. In 3623 patients followed over a 7 years period, higher protein intake did not have a major effect on kidney function decline among elderly men and women [60]. However, because measured creatinine clearance was lower in older *vs.* younger patients in our studies of protein requirements in obese and non-obese critically ill patients [41,42], we examined the impact of protein intake upon serum urea nitrogen concentrations to ascertain the risk for excessive ureagenesis and azotemia.

### 5.2. Ureagenesis and Azotemia in Obese Patients during Hypocaloric High Protein Nutrition Therapy

**Figure 3:**
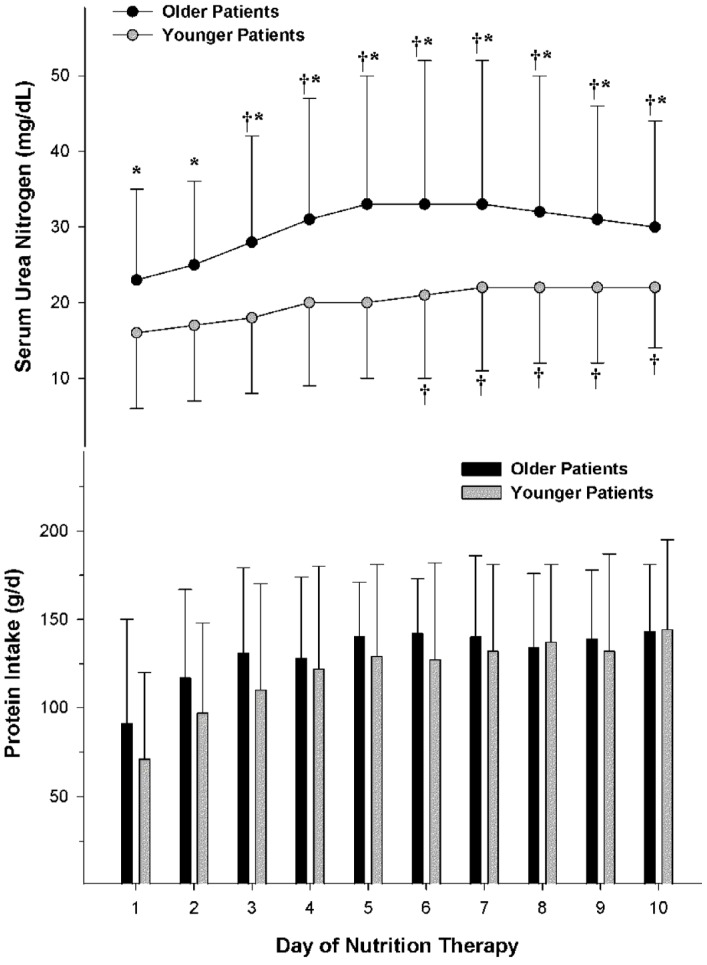
Serum urea nitrogen concentrations and protein intake during hypocaloric, high protein nutrition therapy in older *vs.* younger critically ill patients with obesity and traumatic injuries. Serial serum urea nitrogen concentrations were greater for older compared to younger patients (*p* = 0.001) despite isonitrogenous daily intakes between age groups. * *p* = 0.05 between age groups; † *p* = 0.05 from day 1. Reprinted with permission from Dickerson, R.N.; Medling, T.L.; Smith, A.C.; Maish, G.O., 3rd; Croce, M.A.; Minard, G.; Brown, R.O. Hypocaloric, high-protein nutrition therapy in older *vs.* younger critically ill patients with obesity. *JPEN J. Parenter. Enter. Nutr.* **2013**, *37*, 342–351. A.S.P.E.N. does not endorse the use of this material in any form other than its entirety.

During hypocaloric, high protein nutrition therapy for critically ill patients with obesity, older patients experienced a greater mean serum urea nitrogen concentration than younger patients (30 ± 14 mg/dL *vs.* 20 ± 9 mg/dL, *p* = 0.001), but none of the patients had evidence of renal failure or required hemodialysis or a restriction in protein intake (Figure 3) [42]. Measured creatinine clearance was about 20 mL/min greater than predicted by the Cockcroft-Gault equations [57] for each age group. This exaggerated clearance was likely reflective of the combined effect of availability in renal functional reserve and the hyperdynamic response to critical illness. The statistically significant, but clinically insignificant, difference in serum urea nitrogen concentration between age groups may have been due to multiple factors including achievement of maximal net protein efficiency with excess protein contributing to greater urea production, excessive protein catabolism, an inability to completely compensate for excessive urea production, and/or potential intravascular volume depletion due to diuresis or increased insensible fluid losses [42].

### 5.3. Ureagenesis and Azotemia in Non-Obese Patients

**Figure 4:**
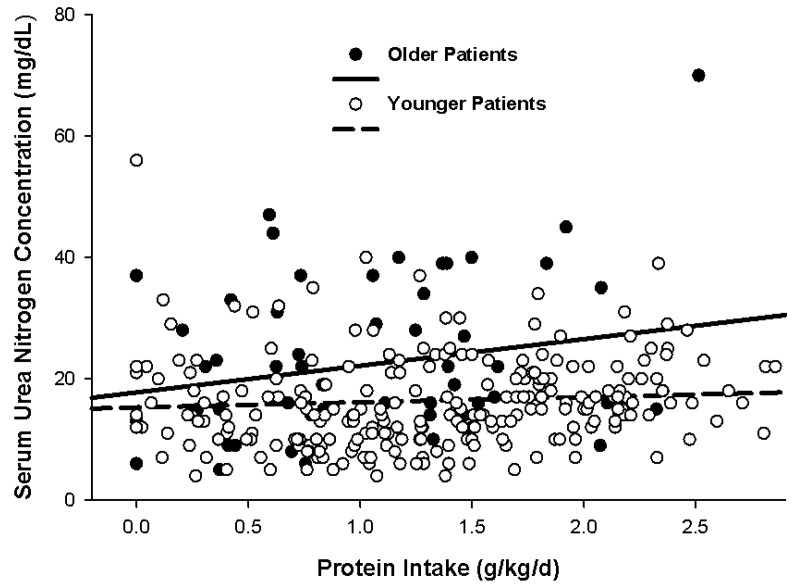
Variability in serum urea nitrogen concentration at different protein intakes. The linear correlative relationship between serum urea nitrogen concentration and protein intake was r = 0.500 (*p* < 0.001) and r = 0.482 (*p* < 0.001) for older and younger patients, respectively. Analysis of covariance indicated no significant difference (*p* = N.S.) in the slopes between the age groups. Reprinted with permission from Dickerson, R.N.; Maish, G.O., 3rd; Croce, M.A.; Minard, G.; Brown, R.O. Influence of aging on nitrogen accretion during critical illness. *JPEN J. Parenter. Enter. Nutr.* **2015**, *39*, 282–290. A.S.P.E.N. does not endorse the use of this material in any form other than its entirety.

Upon examination of ureagenesis in 54 older *vs.* 195 younger non-obese, critically ill patients across a spectrum of protein intake ranging from 0 to 2.8 g/kg/day, there was no significant difference in the rate of rise in serum urea nitrogen concentration between groups (Figure 4) [41]. However, older patients tended to exhibit mildly higher serum urea nitrogen concentrations than younger patients at the same protein intake. Median serum urea nitrogen during the nitrogen balance determination was greater overall for older patients compared with their younger counterparts (20 mg/dL *vs.* 15 mg/dL, *p* = 0.001) despite a lower overall protein intake in the older group (1.1 g/kg/day *vs.* 1.3 g/kg/kg/day) [41]. However, Figure 4 illustrates the wide variability in serum urea nitrogen concentration for both age groups.

Recent data from observational studies suggest a protein intake ≥1.2 g/kg/day during critical illness improves ICU survival when compared to those given lower protein intakes [27,61,62]. Thus, in this author’s opinion, the limitation of protein intake on a short-term basis is unwarranted in the patient without overt acute kidney failure and contraindication for hemodialysis. The whole theoretical point of compromising renal function with higher protein intakes is moot if the patient is not given a sufficient protein intake in an effort to survive the acute insult that led to ICU admission. It is suggested that individualization of protein intake with close monitoring is warranted.

## 6. Higher Protein Dosing without Overfeeding in Older Critically Ill Patients

The loss of fat-free mass associated with aging leads to a loss in a major metabolically active component of the body as there is a reduction in muscle mass [63] and as well as changes in other metabolically active components [64]. Thus, there is an anticipated age-related decline in resting energy expenditure and caloric prescription based on body weight becomes less reliable. In a cohort of healthy elderly subjects, aged 70 to 98 years, the Harris-Benedict equations [65] performed the best in estimation of energy expenditure in comparison to other methods [66]. However, even with use of the Harris-Benedict equations, a broadened error in estimation of energy requirements for hospitalized older patients is expected [67].

Since commercially available enteral formulas have a fixed calorie and protein content, the dilemma faced by clinicians is how to provide sufficient protein intake without giving excessive caloric intake to older critically ill patients. If caloric intake is restricted to 1.2 or 1.3 times the basal energy expenditure (e.g., Harris-Benedict equation), inadequate protein intake may be provided if the patients are given a ~1.0 to 1.06 kcal/mL, 40 to 44 g of protein/L formula. For some highly catabolic patients, even a 1 kcal/mL, 62–64 g of protein/L formula may be insufficient in providing enough protein without providing an excessive caloric intake. A recent observational cohort study in 389 patients examined the influence of how recent guidelines recommending reduced energy targets have resulted in not only a lower energy delivery but also a reduced protein intake (e.g., 81 g/day *vs.* 65 g/day, *p* < 0.0001) [68]. This reduction in enteral feeding intake was associated with prolonged mechanical ventilation (5.0 days *vs.* 6.7 days, *p* = 0.004), extended icu stay (8.5 *vs.* 9.9 days, *p* = 0.0036), and a longer hospital stay (23.4 *vs.* 26.4 days, *p* = 0.028) in survivors. These data give support to the use of concurrent protein supplements, given as bolus doses, in addition to commercially available enteral nutrition formulas in an effort to maximize protein intake while limiting excessive caloric intake for populations such as the elderly whose protein needs may be greater yet caloric needs are reduced. Further research needs implementing regarding the benefit of this nutritional approach.

## 7. Conclusions

Older critically ill patients, especially those with sarcopenia, experience higher mortality and worsened morbidity compared to their younger counterparts. Because older patients have a diminished ability to mount a similar anabolic response to protein supplementation than younger patients during critical illness and, in health, older patients may require a higher protein intake to overcome this “anabolic resistance”. Older critically ill patients tended to have more ureagenesis at similar protein intakes as younger patients, but the difference in serum urea nitrogen concentrations between age groups was not clinically significant for the majority of patients. Randomized controlled trials evaluating the benefits from individualization of protein intake, with close monitoring to avoid potential azotemia, are warranted.

## References

[1] Dickerson R.N., Pitts S.L., Maish G.O., Schroeppel T.J., Magnotti L.J., Croce M.A., Minard G., Brown R.O. (2012). A reappraisal of nitrogen requirements for patients with critical illness and trauma. J. Trauma Acute Care Surg..

[2] Monk D.N., Plank L.D., Franch-Arcas G., Finn P.J., Streat S.J., Hill G.L. (1996). Sequential changes in the metabolic response in critically injured patients during the first 25 days after blunt trauma. Ann. Surg..

[3] Doig G.S., Heighes P.T., Simpson F., Sweetman E.A. (2011). Early enteral nutrition reduces mortality in trauma patients requiring intensive care: A meta-analysis of randomised controlled trials. Injury.

[4] Rapp R.P., Young B., Twyman D., Bivins B.A., Haack D., Tibbs P.A., Bean J.R. (1983). The favorable effect of early parenteral feeding on survival in head-injured patients. J. Neurosurg..

[5] Sacks G.S., Brown R.O., Teague D., Dickerson R.N., Tolley E.A., Kudsk K.A. (1995). Early nutrition support modifies immune function in patients sustaining severe head injury. JPEN J. Parenter. Enter. Nutr..

[6] Hartl R., Gerber L.M., Ni Q., Ghajar J. (2008). Effect of early nutrition on deaths due to severe traumatic brain injury. J. Neurosurg..

[7] Moisey L.L., Mourtzakis M., Cotton B.A., Premji T., Heyland D.K., Wade C.E., Bulger E., Kozar R.A., Nutrition and Rehabilitation Investigators Consortium (NUTRIC) (2013). Skeletal muscle predicts ventilator-free days, ICU-free days, and mortality in elderly ICU patients. Crit. Care.

[8] Du Y., Karvellas C.J., Baracos V., Williams D.C., Khadaroo R.G., Acute Care and Emergency Surgery (ACES) Group (2014). Sarcopenia is a predictor of outcomes in very elderly patients undergoing emergency surgery. Surgery..

[9] Wolfe R.R., Miller S.L., Miller K.B. (2008). Optimal protein intake in the elderly. Clin. Nutr..

[10] Bauer J., Biolo G., Cederholm T., Cesari M., Cruz-Jentoft A.J., Morley J.E., Phillips S., Sieber C., Stehle P., Teta D. (2013). Evidence-based recommendations for optimal dietary protein intake in older people: A position paper from the PROT-Age study group. J. Am. Med. Dir. Assoc..

[11] Flodin L., Svensson S., Cederholm T. (2000). Body mass index as a predictor of 1 year mortality in geriatric patients. Clin. Nutr..

[12] Stevens J., Cai J., Pamuk E.R., Williamson D.F., Thun M.J., Wood J.L. (1998). The effect of age on the association between body-mass index and mortality. N. Engl. J. Med..

[13] Winter J.E., MacInnis R.J., Wattanapenpaiboon N., Nowson C.A. (2014). BMI and all-cause mortality in older adults: A meta-analysis. Am. J. Clin. Nutr..

[14] Al Snih S., Ottenbacher K.J., Markides K.S., Kuo Y.F., Eschbach K., Goodwin J.S. (2007). The effect of obesity on disability *vs.* mortality in older Americans. Arch. Intern. Med..

[15] Potter J.F., Schafer D.F., Bohi R.L. (1988). In-hospital mortality as a function of body mass index: An age-dependent variable. J. Gerontol..

[16] Timmerman K.L., Volpi E. (2008). Amino acid metabolism and regulatory effects in aging. Curr. Opin. Clin. Nutr. Metab. Care.

[17] Cheng A.T., Plank L.D., Hill G.L. (1998). Prolonged overexpansion of extracellular water in elderly patients with sepsis. Arch. Surg..

[18] Luckey A.E., Parsa C.J. (2003). Fluid and electrolytes in the aged. Arch. Surg..

[19] Dennis R.A., Johnson L.E., Roberson P.K., Heif M., Bopp M.M., Cook J., Sullivan D.H. (2008). Changes in prealbumin, nutrient intake, and systemic inflammation in elderly recuperative care patients. J. Am. Geriatr. Soc..

[20] Cruz-Jentoft A.J., Baeyens J.P., Bauer J.M., Boirie Y., Cederholm T., Landi F., Martin F.C., Michel J.P., Rolland Y., Schneider S.M. (2010). Sarcopenia: European consensus on definition and diagnosis: Report of the European Working Group on Sarcopenia in Older People. Age Ageing.

[21] Fried L.P., Tangen C.M., Walston J., Newman A.B., Hirsch C., Gottdiener J., Seeman T., Tracy R., Kop W.J., Burke G. (2001). Frailty in older adults: Evidence for a phenotype. J. Gerontol. A Biol. Sci. Med. Sci..

[22] Chevalier S., Gougeon R., Nayar K., Morais J.A. (2003). Frailty amplifies the effects of aging on protein metabolism: Role of protein intake. Am. J. Clin. Nutr..

[23] Mackenzie T.A., Clark N.G., Bistrian B.R., Flatt J.P., Hallowell E.M., Blackburn G.L. (1985). A simple method for estimating nitrogen balance in hospitalized patients: A review and supporting data for a previously proposed technique. J. Am. Coll. Nutr..

[24] Dickerson R.N., Tidwell A.C., Minard G., Croce M.A., Brown R.O. (2005). Predicting total urinary nitrogen excretion from urinary urea nitrogen excretion in multiple-trauma patients receiving specialized nutrition support. Nutrition.

[25] Shaw-Delanty S.N., Elwyn D.H., Jeejeebhoy K.N., Askanazi J., Schwarz Y., Iles M., Kinney J.M. (1987). Components of nitrogen excretion in hospitalised adult patients on intravenous diets. Clin. Nutr..

[26] Waxman K., Rebello T., Pinderski L., O’Neal K., Khan N., Tourangeau S., Himes E., Cordill K. (1987). Protein loss across burn wounds. J. Trauma.

[27] Allingstrup M.G., Esmailzadeh N., Wilkens Knudsen A., Espersen K., Hartvig Jensen T., Wiis J., Perner A., Kondrup J. (2012). Provision of protein and energy in relation to measured requirements in intensive care patients. Clin. Nutr..

[28] Scheinkestel C.D., Kar L., Marshall K., Bailey M., Davies A., Nyulasi I., Tuxen D.V. (2003). Prospective randomized trial to assess caloric and protein needs of critically ill, anuric, ventilated patients requiring continuous renal replacement therapy. Nutrition.

[29] Campbell W.W., Crim M.C., Dallal G.E., Young V.R., Evans W.J. (1994). Increased protein requirements in elderly people: New data and retrospective reassessments. Am. J. Clin. Nutr..

[30] Morse M.H., Haub M.D., Evans W.J., Campbell W.W. (2001). Protein requirement of elderly women: Nitrogen balance responses to three levels of protein intake. J. Gerontol. A Biol. Sci. Med. Sci..

[31] Volpi E., Mittendorfer B., Rasmussen B.B., Wolfe R.R. (2000). The response of muscle protein anabolism to combined hyperaminoacidemia and glucose-induced hyperinsulinemia is impaired in the elderly. J. Clin. Endocrinol. Metab..

[32] Katsanos C.S., Kobayashi H., Sheffield-Moore M., Aarsland A., Wolfe R.R. (2005). Aging is associated with diminished accretion of muscle proteins after the ingestion of a small bolus of essential amino acids. Am. J. Clin. Nutr..

[33] Volpi E., Mittendorfer B., Wolf S.E., Wolfe R.R. (1999). Oral amino acids stimulate muscle protein anabolism in the elderly despite higher first-pass splanchnic extraction. Am. J. Physiol..

[34] Volpi E., Ferrando A.A., Yeckel C.W., Tipton K.D., Wolfe R.R. (1998). Exogenous amino acids stimulate net muscle protein synthesis in the elderly. J. Clin. Investig..

[35] Rasmussen B.B., Fujita S., Wolfe R.R., Mittendorfer B., Roy M., Rowe V.L., Volpi E. (2006). Insulin resistance of muscle protein metabolism in aging. FASEB J..

[36] Koopman R., Verdijk L., Manders R.J., Gijsen A.P., Gorselink M., Pijpers E., Wagenmakers A.J., van Loon L.J. (2006). Co-ingestion of protein and leucine stimulates muscle protein synthesis rates to the same extent in young and elderly lean men. Am. J. Clin. Nutr..

[37] Katsanos C.S., Kobayashi H., Sheffield-Moore M., Aarsland A., Wolfe R.R. (2006). A high proportion of leucine is required for optimal stimulation of the rate of muscle protein synthesis by essential amino acids in the elderly. Am. J. Physiol. Endocrinol. Metab..

[38] Deutz N.E., Matheson E.M., Matarese L.E., Luo M., Baggs G.E., Nelson J.L., Hegazi R.A., Tappenden K.A., Ziegler T.R., NOURISH Study Group (2016). Readmission and mortality in malnourished, older, hospitalized adults treated with a specialized oral nutritional supplement: A randomized clinical trial. Clin. Nutr..

[39] Bos C., Benamouzig R., Bruhat A., Roux C., Mahé S., Valensi P., Gaudichon C., Ferrière F., Rautureau J., Tomé D. (2000). Short-term protein and energy supplementation activates nitrogen kinetics and accretion in poorly nourished elderly subjects. Am. J. Clin. Nutr..

[40] Guillet C., Prod’homme M., Balage M., Gachon P., Giraudet C., Morin L., Grizard J., Boirie Y. (2004). Impaired anabolic response of muscle protein synthesis is associated with S6K1 dysregulation in elderly humans. FASEB J..

[41] Dickerson R.N., Maish G.O., Croce M.A., Minard G., Brown R.O. (2015). Influence of aging on nitrogen accretion during critical illness. JPEN J. Parenter. Enter. Nutr..

[42] Dickerson R.N., Medling T.L., Smith A.C., Maish G.O., Croce M.A., Minard G., Brown R.O. (2013). Hypocaloric, high-protein nutrition therapy in older *vs.* younger critically ill patients with obesity. JPEN J. Parenter. Enter. Nutr..

[43] Liu K.J., Cho M.J., Atten M.J., Panizales E., Walter R., Hawkins D., Donahue P.A. (2000). Hypocaloric parenteral nutrition support in elderly obese patients. Am. Surg..

[44] Choban P., Dickerson R., Malone A., Worthington P., Compher C., American Society for Parenteral and Enteral Nutrition (2013). A.S.P.E.N. Clinical guidelines: Nutrition support of hospitalized adult patients with obesity. JPEN J. Parenter. Enter. Nutr..

[45] McClave S.A., Taylor B.E., Martindale R.G., McCarthy M., Roberts P., Taylor B., Ochoa J.B., Napolitano L., Cresci G., A.S.P.E.N. Board of Directors (2016). Guidelines for the Provision and Assessment of Nutrition Support Therapy in the Adult Critically Ill Patient: Society of Critical Care Medicine (SCCM) and American Society for Parenteral and Enteral Nutrition (A.S.P.E.N.). JPEN J. Parenter. Enter. Nutr..

[46] Dickerson R.N., Boschert K.J., Kudsk K.A., Brown R.O. (2002). Hypocaloric enteral tube feeding in critically ill obese patients. Nutrition.

[47] Dickerson R.N., Rosato E.F., Mullen J.L. (1986). Net protein anabolism with hypocaloric parenteral nutrition in obese stressed patients. Am. J. Clin. Nutr..

[48] Choban P.S., Dickerson R.N. (2005). Morbid obesity and nutrition support: Is bigger different?. Nutr. Clin. Pract..

[49] Dickerson R.N., Mason D.L., Croce M.A., Minard G., Brown R.O. (2005). Evaluation of an artificial neural network to predict urea nitrogen appearance for critically ill multiple-trauma patients. JPEN J. Parenter. Enter. Nutr..

[50] Kaysen G.A., Myers B.D. (1985). The aging kidney. Clin. Geriatr. Med..

[51] Lindeman R.D., Tobin J., Shock N.W. (1985). Longitudinal studies on the rate of decline in renal function with age. J. Am. Geriatr. Soc..

[52] Fliser D., Zeier M., Nowack R., Ritz E. (1993). Renal functional reserve in healthy elderly subjects. J. Am. Soc. Nephrol..

[53] Lew S.W., Bosch J.P. (1991). Effect of diet on creatinine clearance and excretion in young and elderly healthy subjects and in patients with renal disease. J. Am. Soc. Nephrol..

[54] Bosch J.P., Saccaggi A., Lauer A., Ronco C., Belledonne M., Glabman S. (1983). Renal functional reserve in humans. Effect of protein intake on glomerular filtration rate. Am. J. Med..

[55] Klahr S., Levey A.S., Beck G.J., Caggiula A.W., Hunsicker L., Kusek J.W., Striker G. (1994). The effects of dietary protein restriction and blood-pressure control on the progression of chronic renal disease. Modification of Diet in Renal Disease Study Group. N. Engl. J. Med..

[56] Levey A.S., Greene T., Sarnak M.J., Wang X., Beck G.J., Kusek J.W., Collins A.J., Kopple J.D. (2006). Effect of dietary protein restriction on the progression of kidney disease: Long-term follow-up of the Modification of Diet in Renal Disease (MDRD) Study. Am. J. Kidney Dis..

[57] Cockcroft D.W., Gault M.H. (1976). Prediction of creatinine clearance from serum creatinine. Nephron.

[58] Fliser D., Bischoff I., Hanses A., Block S., Joest M., Ritz E., Mutschler E. (1999). Renal handling of drugs in the healthy elderly. Creatinine clearance underestimates renal function and pharmacokinetics remain virtually unchanged. Eur. J. Clin. Pharmacol..

[59] Fliser D., Ritz E. (2001). Serum cystatin C concentration as a marker of renal dysfunction in the elderly. Am. J. Kidney Dis..

[60] Beasley J.M., Katz R., Shlipak M., Rifkin D.E., Siscovick D., Kaplan R. (2014). Dietary protein intake and change in estimated GFR in the Cardiovascular Health Study. Nutrition.

[61] Nicolo M., Heyland D.K., Chittams J., Sammarco T., Compher C. (2016). Clinical Outcomes Related to Protein Delivery in a Critically Ill Population: A Multicenter, Multinational Observation Study. JPEN J. Parenter. Enter. Nutr..

[62] Weijs P.J., Stapel S.N., de Groot S.D., Driessen R.H., de Jong E., Girbes A.R., van Schijndel R.J.S., Beishuizen A. (2012). Optimal protein and energy nutrition decreases mortality in mechanically ventilated, critically ill patients: A prospective observational cohort study. JPEN J. Parenter. Enter. Nutr..

[63] Zurlo F., Larson K., Bogardus C., Ravussin E. (1990). Skeletal muscle metabolism is a major determinant of resting energy expenditure. J. Clin. Investig..

[64] Bosy-Westphal A., Eichhorn C., Kutzner D., Illner K., Heller M., Muller M.J. (2003). The age-related decline in resting energy expenditure in humans is due to the loss of fat-free mass and to alterations in its metabolically active components. J. Nutr..

[65] Harris J.A., Benedict F.G. (1918). A Biometric Study of Human Basal Metabolism. Proc. Natl. Acad. Sci. USA.

[66] Melzer K., Laurie Karsegard V., Genton L., Kossovsky M.P., Kayser B., Pichard C. (2007). Comparison of equations for estimating resting metabolic rate in healthy subjects over 70 years of age. Clin. Nutr..

[67] Neelemaat F., van Bokhorst-de van der Schueren M.A., Thijs A., Seidell J.C., Weijs P.J. (2012). Resting energy expenditure in malnourished older patients at hospital admission and three months after discharge: Predictive equations *versus* measurements. Clin. Nutr..

[68] Berger M.M., Soguel L., Charriere M., Theriault B., Pralong F., Schaller M.D. (2016). Impact of the reduction of the recommended energy target in the ICU on protein delivery and clinical outcomes. Clin. Nutr..

